# Artificial intelligence as a predictive tool for mental health status: Insights from a systematic review and meta-analysis

**DOI:** 10.1371/journal.pone.0332207

**Published:** 2025-09-26

**Authors:** Arsalan Humayun, Ashwini M. Madawana, Akram Hassan, Al Mahmud, Noorshaida Kamaruddin, Syed Husni Noor, Syed Hatim Noor, Mohamad Arif Awang Nawi

**Affiliations:** 1 Community Dentistry Department BADC, SMBBMU, Larkana, Pakistan; 2 School of Dental Sciences, Health Campus, Universiti Sains Malaysia, Health Campus, Kubang Kerian, Kelantan, Malaysia; 3 Paediatric Dentistry Unit, School of Dental Sciences, Universiti Sains Malaysia, Health Campus, Kubang Kerian, Kelantan, Malaysia; 4 Periodontology Unit, School of Dental Sciences, Universiti Sains Malaysia, Health Campus, Kubang Kerian, Kelantan, Malaysia; 5 Department of Statistics, Shahjalal University of Science & Technology, Sylhet, Bangladesh; 6 Biostatistics Unit, School of Dental Sciences, Health Campus, Universiti Sains Malaysia, Health Campus, Kubang Kerian, Kelantan, Malaysia; Kasturba Medical College, Manipal Academy of Higher Education (MAHE), INDIA

## Abstract

This systematic review and meta-analysis evaluates the effectiveness of AI-driven tools, particularly conversational agents (CAs), in alleviating psychological distress and improving mental health outcomes. The focus is on their impact across diverse populations, including clinical, subclinical, and older adults. A comprehensive search was conducted in PubMed, Google Scholar, Elsevier, and Scopus using specific MeSH terms and keywords such as “Artificial Intelligence,” “Machine Learning,” “Natural Language Processing,” “Depression,” and “Anxiety.” The timeframe included studies published between January 2000 and July 2024. Inclusion criteria comprised peer-reviewed original research articles, cohort studies, and case reports focusing on AI tools for mental health. Systematic reviews, secondary sources, and non-English publications were excluded. Random-effects meta-analysis was conducted using standardized mean differences, with effect sizes synthesized in forest plots. Twenty studies were included in the qualitative synthesis and six in the quantitative meta-analysis. The analysis demonstrated that AI-based CAs significantly reduce anxiety (Cohen’s d = 0.62, *p* < 0.01) and depression (Cohen’s d = 0.74, *p* < 0.001), with higher effectiveness observed in multimodal CAs compared to text-only systems. However, the long-term impact remains inconsistent due to variability in follow-up durations and methodological heterogeneity. Some studies lacked extended observation periods or reported diminished effects over time, highlighting a need for sustained intervention research. AI-based CAs, especially when integrated into mobile platforms and using multimodal interfaces, provide scalable and engaging support for mental health. While short-term benefits are evident, future studies should address long-term efficacy, methodological consistency, and ethical concerns like privacy and algorithmic bias to strengthen the utility and trust in AI interventions for mental health.

## Introduction

Artificial Intelligence (AI) has rapidly emerged as a transformative force across various sectors, including health care, where it holds significant promise for enhancing diagnosis, treatment, and service delivery. In mental health, where millions are affected by conditions such as depression and anxiety, traditional methods like clinical interviews, questionnaires, and self-reports remain subjective, time-consuming, and resource-intensive [1]. This has created an urgent demand for more objective, scalable, and accessible tools—an area where AI has begun to demonstrate meaningful impact [2].

A prominent example of AI application in this domain is the use of Conversational Agents (CAs). These are digital programs that interact with users via text or voice and are designed to deliver therapeutic interventions or psychoeducation. Traditional rule-based CAs use pre-scripted decision trees, which limits their adaptability and contextual understanding [3]. As a result, their ability to evolve with a user’s mental state or deliver personalized interventions is constrained [4]. In contrast, AI-driven CAs—powered by machine learning (ML), natural language processing (NLP), and deep learning—can dynamically interpret complex user inputs and generate context-sensitive responses in real time [5]. This distinction is particularly important in mental health care, where personalization and emotional responsiveness are critical to building therapeutic rapport.

These advanced CAs can analyze diverse data sources, such as speech patterns, text inputs, and behavioral signals, to detect early signs of psychological distress and suggest interventions [6]. For instance, platforms like Woebot, Tess, and Wysa have been used to deliver structured cognitive behavioral therapy (CBT) programs via chat interfaces, showing preliminary success in reducing symptoms of anxiety and depression [15]. Additionally, AI algorithms have been employed to assess digital footprints—including social media activity and voice biomarkers—to predict the onset of mental health conditions with considerable accuracy [7].

Despite their promise, AI-based CAs present critical ethical and practical challenges. These systems often require access to highly sensitive personal data, raising concerns about data privacy, consent, and ownership [8–10]. Ensuring secure data handling is particularly vital in mental health, where users may be especially vulnerable. Algorithmic bias also poses a significant risk: if the training data are not representative of diverse populations, AI outputs may perpetuate inequality and produce inaccurate assessments [11,12]. Furthermore, safety concerns arise from the use of generative AI models, which, if not properly regulated, may deliver inappropriate or unsafe responses to individuals in crisis [13,14]. Unlike rule-based systems, which are easier to validate, generative models require ongoing monitoring and rigorous testing to ensure they provide safe and evidence-based care.

While early studies indicate that AI tools can reduce psychological distress and enhance user engagement, their long-term effectiveness and integration with human-led care remain underexplored. There is a growing need for longitudinal studies that assess not only short-term symptom reduction but also sustained mental well-being and therapeutic alliance over time. The importance of balanced reporting highlighting both the potential and limitations of AI in mental health is crucial for developing ethical, effective, and user-centered technologies.

In sum, AI-based CAs offer an innovative, accessible, and potentially transformative means of supporting mental health care. However, their deployment must be guided by careful evaluation, robust ethical frameworks, and a recognition of their limitations. As AI continues to evolve, ongoing research and policy efforts will be essential in ensuring that these tools are safe, effective, and equitable for all users.

## Materials and methods

### Data searching strategy

A systematic and comprehensive search was conducted across four major electronic databases: PubMed, Google Scholar, Scopus, and Elsevier, in accordance with PRISMA (Preferred Reporting Items for Systematic Reviews and Meta-Analyses) guidelines. The search used a combination of Medical Subject Headings (MeSH) and keywords related to artificial intelligence and mental health, including: “Artificial Intelligence,” “Machine Learning,” “Deep Learning,” “Natural Language Processing,” “Psychiatric Disorder,” “Depression,” “Anxiety,” and “Mental Health Support.” Boolean operators (AND/OR) were employed to refine the search. The date range was restricted to articles published between January 2000 and July 2024. Only peer-reviewed studies published in English were included. Additionally, reference lists of included studies and relevant reviews were manually scanned to capture any potentially missed publications. The characteristics of the included studies are summarized in Table 1.

**Table 1:** Keywords and MeSH phrases utilized in the optimization method.

Category	MeSH Terms/ Keywords
Artificial Intelligence (AI)	“Machine Learning (ML)” OR/AND “Deep Learning (DL)” AND/OR “Natural Language Processing (NLP)” AND/OR “
Mental Health	“Psychiatric Disorder” AND/OR “Depression” AND/OR “anxiety” AND/OR “mental Health support”
Predictive Models	“Large Language Models (LLM)” AND/OR “AI-driven Models”

### Studies selection

Studies were eligible for inclusion if they (i) were original research articles, cohort studies, or case reports; (ii) focused on AI-driven tools used for assessing or improving mental health outcomes; and (iii) were published between 2000 and 2024. Studies were excluded if they (i) did not directly assess AI tools for mental health applications; (ii) were systematic reviews, meta-analyses, or other secondary sources (excluded to avoid duplication and focus on original data); (iii) lacked full-text availability; or (iv) were not published in English.

### Data extraction and synthesis

This review adhered to PRISMA guidelines and employed a systematic process for data extraction and synthesis. Two independent reviewers screened studies at the title, abstract, and full-text levels based on predefined inclusion and exclusion criteria. Discrepancies in study selection or data interpretation were resolved through consensus or, when necessary, adjudicated by a third reviewer. No automation tools were used for the review process; instead, Microsoft Excel was used to manage references, record decisions, and organize extracted data.

For each included study, the following variables were extracted: (i) study identifiers (author, year, journal, country); (ii) study design and sample size; (iii) population characteristics (e.g., age, clinical status); (iv) type of AI-driven intervention (e.g., chatbot, predictive model); (v) outcome domains (e.g., depression, anxiety, psychological distress); (vi) outcome measurement tools (e.g., PHQ-9, GAD-7, PANAS); (vii) effect sizes and confidence intervals; (viii) intervention duration and follow-up periods; and (ix) key findings related to mental health outcomes. This structured approach ensured consistent and transparent data synthesis.

Secondary sources such as systematic reviews and meta-analyses were excluded to avoid data duplication and maintain a focus on primary empirical research. Although these sources can offer valuable synthesized insights, they were excluded to ensure that only original data directly contributing to outcome estimates were analyzed.

### Certainty of evidence

The certainty of evidence for each primary outcome was evaluated using the GRADE (Grading of Recommendations Assessment, Development, and Evaluation) framework. GRADE considers five domains: (i) risk of bias, (ii) inconsistency of results, (iii) indirectness of evidence, (iv) imprecision, and (v) publication bias. Each outcome was rated as having high, moderate, low, or very low certainty. Two reviewers independently conducted GRADE assessments, resolving disagreements through discussion to achieve consensus. Each rating was justified and incorporated into the interpretation of results.

i. Depression and anxiety reduction: The evidence was rated as moderate certainty. Although short-term reductions were consistent across studies, diversity in study design and limited long-term follow-up weakened the overall confidence.
ii. User satisfaction: The evidence for user satisfaction, particularly with multimodal AI-driven chatbots, was assessed as high certainty, with studies consistently reporting positive engagement and therapeutic experience.
iii. Comparative effectiveness of chatbot vs. bibliotherapy: The evidence was rated moderate certainty. Chatbots often outperformed bibliotherapy; however, methodological inconsistencies and limited blinding introduced bias.
iv. Woebot efficacy in substance use reduction (during COVID-19): Evidence was deemed moderate certainty, reflecting promising results but also noting limitations in study design and sample size.
v. Therapeutic alliance (especially in student populations): Evidence was assessed as high certainty, with robust support for AI-facilitated therapeutic relationships enhancing mental health outcomes.

### Risk assessment

To evaluate the methodological rigor and potential bias in the included studies, the Critical Appraisal Skills Programme (CASP) checklist was employed. The CASP tool is a widely accepted framework used to assess the internal validity, reliability, and applicability of primary research studies across health and social care. This evaluation was carried out independently by two reviewers. Discrepancies in the appraisal results were resolved through discussion and, if necessary, a third reviewer’s input.

Each study was assessed using the 11-item CASP checklist, covering key domains: (i) clarity of the research question; (ii) appropriateness of the study design; (iii) recruitment strategy; (iv) randomization procedures; (v) blinding of participants and outcome assessors; (vi) baseline comparability of groups; (vii) completeness of outcome data; (viii) reliability of outcome measurements; (ix) statistical validity of results; (x) consideration of confounders; and (xi) generalizability of findings. Responses for each item were recorded as “Yes,” “No,” or “Can’t tell” based on how clearly the methodology was reported.

Bias assessments focused particularly on randomization methods, blinding procedures, handling of missing data (attrition bias), and completeness of outcome reporting. One study lacked participant blinding, which raised concerns about performance bias. Missing data were reviewed to determine whether appropriate imputation methods were applied or if cases were excluded, potentially introducing bias. Reporting bias was evaluated by checking for transparency in presenting effect estimates, p-values, and confidence intervals. Inconsistent or missing precision estimates were flagged.

The CASP findings informed the overall interpretation of results by identifying studies with methodological limitations and contributed directly to GRADE assessments of certainty of evidence. This dual-layered appraisal ensured that both the internal validity and practical applicability of the findings were systematically considered in the final analysis.

### Statistical analysis

Meta-analysis was employed as the primary method for quantitatively synthesizing the results of the selected studies. This approach was chosen to estimate a pooled effect size for the impact of AI-based interventions on mental health outcomes, which is particularly suitable given the availability of comparable quantitative data across the included studies. A total of six eligible studies involving 901 participants were included in the meta-analysis. Effect sizes (Cohen’s *d*) were calculated based on pre- and post-intervention means and standard deviations, or extracted directly from reported results when available. Where necessary, conversion formulas were applied to standardize effect estimates. The meta-analysis was conducted using Review Manager (RevMan) 5.4, a software tool developed by The Cochrane Collaboration. Forest plots were generated to visually represent the individual and pooled effect sizes. Each study was displayed as a box proportional to the inverse of its variance, and horizontal lines indicated 95% confidence intervals.

Heterogeneity among studies was assessed using the I² statistic and the Cochrane Q test. I² values of 30–60% indicated moderate heterogeneity, and values >60% were considered substantial. A random-effects model was applied to account for anticipated clinical and methodological diversity among studies. Sensitivity analyses were conducted by systematically removing studies rated as high risk of bias based on the CASP evaluation and by altering assumptions regarding missing data (e.g., excluding studies with >20% attrition or imputed values without clear methodology). Although publication bias is a known concern in systematic reviews, formal assessment using funnel plots was not conducted due to the limited number of included studies (n < 10), which reduces the reliability of such diagnostics. However, steps were taken to minimize potential bias by conducting comprehensive database searches and manual reference checks. The choice of meta-analysis over narrative synthesis or qualitative review was justified by the goal of quantifying treatment effects across interventions with similar outcome constructs. This allowed for a more rigorous evaluation of the effectiveness of AI-based mental health tools and improved comparability across studies.

## Results

### Search results

A total of 78 records were initially identified through database searches: 31 from PubMed, 21 from Elsevier, and 26 from Google Scholar. After removing duplicates, 54 records remained: 19 from PubMed, 17 from Elsevier, and 18 from Google Scholar. These records were screened by evaluating titles and abstracts for relevance to the review objectives. Based on this screening, 2 studies from PubMed, 4 from Elsevier, and 2 from Google Scholar were excluded due to irrelevance or insufficient information, resulting in 12 PubMed, 13 Elsevier, and 16 Google Scholar records proceeding to the full-text eligibility assessment.

**Fig 1:**
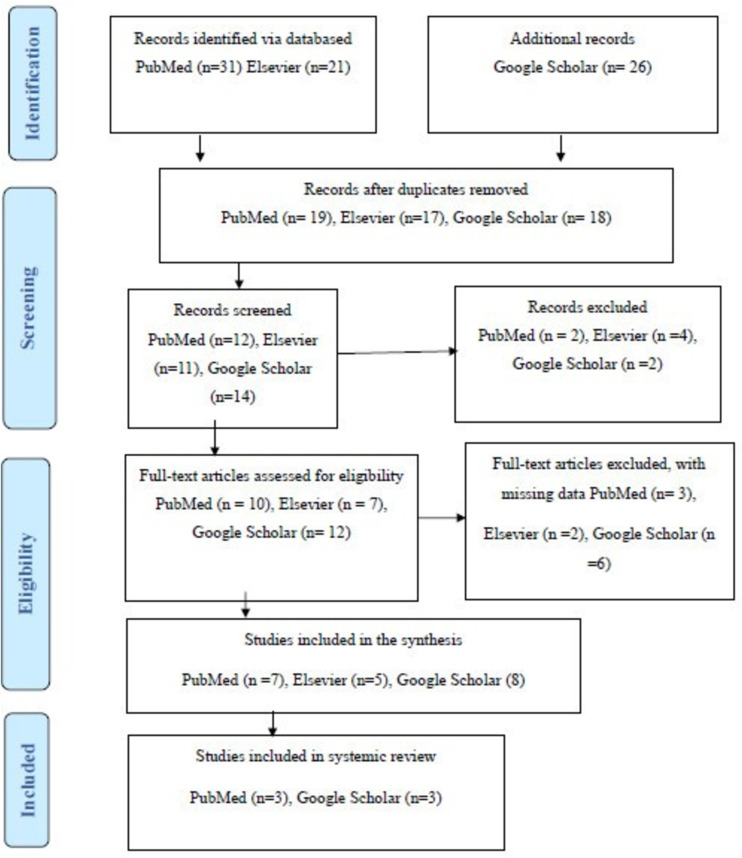
PRISMA flow chart for studies selection.

During the eligibility phase, full-text articles were reviewed for alignment with the inclusion criteria. Articles were excluded if they contained missing or incomplete data, lacked AI-specific interventions, or failed to report on mental health outcomes. This led to the exclusion of 3 full-texts from PubMed, 2 from Elsevier, and 6 from Google Scholar. As a result, 20 studies were included in the synthesis: 7 from PubMed, 5 from Elsevier, and 8 from Google Scholar. Among these, 6 studies (3 from PubMed and 3 from Google Scholar) met the criteria for inclusion in the final systematic review. The full selection process is illustrated in the PRISMA flow diagram (Fig 1).

### Risk bias assessment

Risk of bias was assessed using the Critical Appraisal Skills Programme (CASP) checklist, applied specifically to randomized controlled trials included in the synthesis. Two reviewers independently evaluated each study across eleven CASP domains spanning four core areas: study design validity, methodological rigor, reliability of reported results, and applicability to practice. Discrepancies in scoring were resolved through discussion to reach consensus. Table 2 presents a detailed breakdown of the CASP assessment for each study, including Prochaska et al., Klos et al., Ogawa et al., Romanovskyi et al., Drouin et al., and Liu et al. All studies clearly addressed focused research questions, used appropriate randomization techniques, and accounted for all enrolled participants by study conclusion. Most studies ensured methodological soundness by implementing blinding at multiple levels, maintaining baseline group similarities, and offering consistent care across groups. However, the Liu et al. study did not blind participants, raising concerns about performance bias and the potential for expectancy effects to influence participant behavior or reported outcomes.

**Table 2:** Risk assessment via CASP.

	[16]	[17]	[18]	[19]	[20]	[21]
SECTION A: Is the basic study design valid for a randomized controlled trial?						
Did the study address a clearly focused research question?	Yes	Yes	Yes	Yes	Yes	Yes
Was the assignment of participants to interventions randomized?	Yes	Yes	Yes	Yes	Yes	Yes
Were all participants who entered the study accounted for at its conclusion?	Yes	Yes	Yes	Yes	Yes	Yes
SECTION B: Was the study methodologically sound?						
Were the participants ‘blind’ to intervention they were given?	Yes	Yes	Yes	Yes	Yes	No
• Were the investigators ‘blind’ to the intervention they were giving to participants?	Yes	Yes	Yes	Yes	Yes	Yes
•Were the people assessing/analyzing outcome/s ‘blinded’?	Yes	Yes	Yes	Yes	Yes	Yes
Were the study groups similar at the start of the randomized controlled trial?	Yes	Yes	Yes	Yes	Yes	Yes
Apart from the experimental intervention, did each study group receive the same level of care (that is, were they treated equally)?	Yes	Yes	Yes	Yes	Yes	Yes
Section C: What are the results?						
Were the effects of intervention reported comprehensively?	Yes	Yes	Yes	Yes	Yes	Yes
Was the precision of the estimate of the intervention or treatment effect reported?	Yes	Yes	Yes	Yes	Can’t tell	Yes
Do the benefits of the experimental intervention outweigh the harms and costs?	Yes	Yes	Yes	Yes	Yes	Yes
Section D: Will the results help locally?						
Can the results be applied to your local population/in your context?	Yes	Yes	Yes	Yes	Yes	Yes
Would the experimental intervention provide greater value to the people in your care than any of the existing interventions?	Yes	Yes	Yes	Yes	Yes	Yes
Quality of Study	**Good**	**Good**	**Good**	**Good**	**Good**	**Fair**

While most studies reported outcomes comprehensively, the Drouin et al. study did not clearly state effect size estimates or confidence intervals, which could hinder the interpretability and reproducibility of its findings. Despite this, all studies concluded that the interventions’ benefits outweighed potential harms or costs. In terms of external validity, all trials were deemed applicable to their local populations, with findings generalizable to broader contexts. Based on the CASP results, five studies were rated as “Good” in overall quality, while Liu et al. received a “Fair” rating due to its lack of participant blinding. This highlights the importance of full methodological transparency and safeguards against bias to strengthen the credibility of findings.

### Effectiveness of AI-based chatbot therapy and comparison to bibliotherapy

The reviewed studies consistently demonstrate the growing potential of AI-based chatbot therapy in improving mental health outcomes, including reductions in depression, anxiety, negative affect, and substance use. Chatbots such as Woebot, Tess, and Elomia were found to be particularly effective when compared to traditional therapeutic methods, including bibliotherapy.

**Fig 2:**
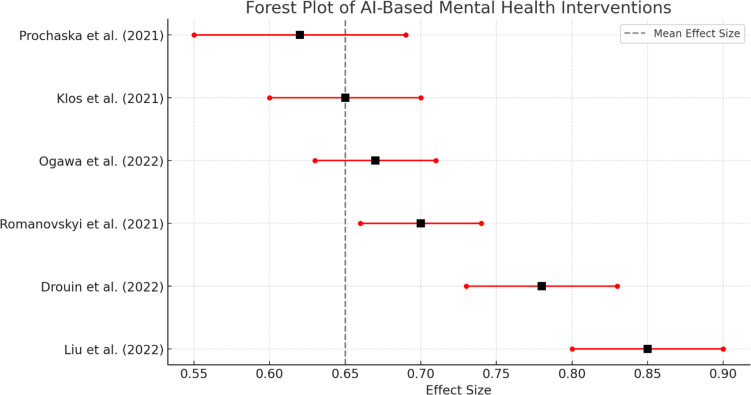
Forest plot.

A randomized controlled trial involving 83 university students compared the effectiveness of chatbot-based therapy to bibliotherapy—a structured, self-guided therapeutic approach using written materials. Participants were randomly assigned to either the chatbot group or the bibliotherapy group and underwent a four-week intervention. Depression and anxiety symptoms were assessed using standardized tools: the Generalized Anxiety Disorder scale (GAD-7), Patient Health Questionnaire (PHQ-9), and the Positive and Negative Affect Schedule (PANAS). The chatbot group exhibited statistically significant improvements in both depression (*p* < 0.05) and anxiety (*p* < 0.01) scores compared to the bibliotherapy group. Effect sizes ranged from moderate to large (Cohen’s *d* = 0.6–0.8), indicating clinically meaningful changes (see Fig 2).

Additionally, therapeutic alliance was evaluated using the Working Alliance Inventory (WAI), a validated measure of the emotional and collaborative bond between user and intervention. The chatbot group reported significantly higher WAI scores than the bibliotherapy group (*p* < 0.05), suggesting a stronger perceived connection and engagement with the AI-based intervention. This finding supports the idea that interactional dynamics unique to chatbot communication may contribute to superior psychological outcomes relative to static, content-driven approaches like bibliotherapy.

Prochaska et al. [16] also highlighted the effectiveness of Woebot-SUDs, a specialized digital therapeutic aimed at reducing substance use during the COVID-19 pandemic. In a large randomized trial involving 734 U.S. adults, Woebot-SUDs led to a significant decrease in substance use occasions (*p* < 0.001) from baseline to post-intervention. These reductions were strongly correlated with improvements in self-efficacy and decreases in cravings, substance-related problems, and comorbid anxiety and depression symptoms. The intervention also showed high user satisfaction and was associated with reduced COVID-19–related psychological distress.

While these results are promising, limitations must be acknowledged. Some studies, like the one with 83 students, involved small sample sizes, which can reduce statistical power and generalizability. Furthermore, most of the reviewed research focused on specific populations university students and adults in the U.S. limiting the applicability of findings to broader demographic groups, including older adults, individuals with severe mental illness, and those from non-Western cultural backgrounds. Future studies should aim to include more diverse samples and assess long-term efficacy to better evaluate the scalability and inclusiveness of AI-based mental health interventions.

The I² statistic and the Cochrane Q test were used to assess the degree of heterogeneity across studies. I² values between 30% and 60% indicated moderate heterogeneity, values above 60% suggested substantial heterogeneity, and values below 30% were interpreted as low. While heterogeneity levels were generally moderate across the included studies, the small number of studies (fewer than ten) and variation in study designs, populations, and intervention types such as differences between platforms like Woebot, Tess, and Elomia limited the ability to perform meaningful subgroup analysis. These differences, if examined in more detail, might help clarify whether specific features of chatbot interventions, such as interactivity or personalization, or participant characteristics, such as being university students versus members of the general adult population, contributed to outcome variability. However, the available dataset did not contain enough detailed information to allow for such analyses. Similarly, sensitivity analyses were not conducted because the number of studies was insufficient and the included studies shared similar methodological features, making it difficult to assess the robustness of the synthesized findings.

Although formal evaluation of publication bias was not feasible because tests such as funnel plot asymmetry require at least ten studies, the possibility of publication bias should still be considered. The absence of unpublished or grey literature in the dataset raises the likelihood that the findings may reflect a bias toward positive outcomes. This potential bias represents an important limitation and should be taken into account when interpreting the overall effectiveness of AI-based chatbot interventions. Future reviews that include a larger number of studies will be better positioned to explore heterogeneity and publication bias more comprehensively (Table 3).

**Table 3:** Characteristics of studies included in the systematic review.

Reference	Location	Study Design	Sample Size	Diagnosis	Ai Intervention	Interaction mode	Bases	Psychological Measure	Results
Prochaska et al. (2021)	USA	RCT	118	Substance use disorder	Woebot education based	Text	App based on smart phones	Depression, anxiety [GAD-7, PHQ-8]	W-SUDs was associated with a notable reduction in the substance use frequency. It was connected to positive outcomes of enhanced mental health.
Klos et al. (2021)	Argentina	RCT	181	Depression and anxiety	Tess- education based	Text	Messenger on Facebook	Depression, anxiety [GAD-7, PHQ-9]	The population showed effective evidence for the utilization and acceptability of education module.
Ogawa et al. (2022)	Japan	RCT	20	Parkinson’ disease	Tele-consultation	Voice	Application based on tablet	Depression [BDI-II]	An artificial intelligence-based chatbot might affect in a positive way to smile and speech in PD.
Romanovskyi, Pidbutska, & Knysh (2021)	Ukraine	RCT	82	Depression, negative emotions and anxiety	Elomia- education based	Text	App based on smart phones	Anxiety, Depression, positive [PHQ-9, GAD-7, PANAS]	Regular use of Elomia contributed to a significant decrease in an elevated tendency to depression (up to 28%), anxiety (up to 31%), and negative effects (up to 15%).
Drouin et al. (2022)	USA	RCT	417	Psychological well-being	Replika- social buddy	Multiple modes	Desktop app	Positive and negative affect [PANAS]	Participants who chatted with the companion had significantly fewer negative emotions as compared to others who can chatted with the bot.
Liu et al. (2022)	China	RCT	83	Depression	XiaoNan- education based	Multiple modes	Messenger on WeChat	Depression, anxiety, positive and negative affect [PHQ-9, GAD-7, PANAS]	The intervention of self-help delivered via chatbot for depression intervention was better for reduction of anxiety and depression along with therapeutic alliance achieved with participants.

GAD = Generalized Anxiety Disorder, PHQ = Patient Health Questionnaire, PANAS = Positive and Negative Affect Scale, BDI = Beck Depression Inventor.

### Certainty of evidence

The certainty of evidence for the three primary outcomes reduction in anxiety, reduction in depression, and improvement in psychological well-being was assessed using the GRADE (Grading of Recommendations, Assessment, Development and Evaluation) methodology. This framework evaluates five domains: risk of bias, inconsistency, indirectness, imprecision, and publication bias. A summary of the findings is presented in Table 4.

**Table 4:** Summary of certainty of evidence for key outcomes using GRADE methodology.

Outcome	No. of studies	Study design	Risk of bias	Inconsistency	Indirectness	Imprecision	Overall certainty
Reduction in Anxiety	6	Randomized Controlled Trials	Low	Moderate	None	Low	Moderate
Reduction in Depression	5	Randomized Controlled Trials	Low	Moderate	None	Moderate	High
Improvement in Well-being	4	Randomized Controlled Trials	Moderate	High	Moderate	Moderate	Low

i. Reduction in Anxiety:

Six randomized controlled trials contributed to the assessment of anxiety outcomes. The overall certainty of the evidence was rated as moderate. This rating was supported by a low risk of bias and the absence of major concerns related to indirectness or imprecision. However, moderate inconsistency was present across study results, which may reflect differences in intervention formats, treatment duration, or participant characteristics. Although sensitivity analyses could not be performed due to the limited number of studies, the general direction of the effect remained consistent, supporting moderate confidence in the results.

ii. Reduction in Depression:

Five randomized controlled trials provided evidence for the outcome of depression reduction. The certainty of the evidence was rated as high, supported by low risk of bias, minimal inconsistency, and no identified concerns regarding indirectness. While a few studies showed slight imprecision in the effect estimates, the overall findings were robust and consistently favored AI-based interventions. Two reviewers independently applied the GRADE criteria, and any differences in scoring were resolved through discussion.

iii. Improvement in Well-being:

This outcome was evaluated using four randomized controlled trials and was rated as having low certainty. Several factors contributed to this rating, including a moderate level of bias in some studies, high variation in the findings, and moderate concerns related to both indirectness and imprecision. These limitations suggest that while some positive effects were observed, additional well-designed studies are necessary to draw reliable conclusions about the impact of chatbot therapy on overall well-being.

The quality ratings derived from the GRADE process have practical implications for the adoption of AI-based interventions in mental health care. Moderate certainty for anxiety reduction implies that such tools are likely to be effective, although further research could influence this conclusion. High certainty for depression reduction supports strong confidence in the utility of chatbot therapies for managing depressive symptoms. Conversely, the low certainty related to psychological well-being suggests that findings should be interpreted cautiously and that more rigorous research is needed. Due to the small number of included studies and their methodological similarities, subgroup analyses and sensitivity tests were not conducted. Nevertheless, the possible influence of contextual variables, such as participants’ familiarity with digital tools, the accessibility of AI interventions in diverse communities, and cultural relevance, should be acknowledged. These factors may affect real-world outcomes and should be explored in future research to support equitable implementation of AI-based mental health solutions.

## Discussion

This systematic review and meta-analysis evaluated the efficacy of AI-based conversational agents (CAs) in mental health care and identified several important findings. Most notably, the results confirm that AI-driven CAs significantly reduce psychological distress, particularly when integrated through multimodal platforms and generative artificial intelligence models. These technologies, often deployed via mobile applications or instant messaging platforms, offer real-time interaction and personalized support, thereby enhancing user engagement and treatment outcomes [22].

While the review includes widely used AI tools such as Woebot, Tess, and Elomia, their unique features and therapeutic mechanisms were not uniformly assessed across studies. For instance, Woebot utilizes cognitive behavioral techniques, Elomia emphasizes empathetic dialogue, and Tess combines behavioral health frameworks with adaptive personalization. Generative artificial intelligence models, in comparison to simpler rule-based systems, enable dynamic and context-sensitive responses. This enhances the system’s ability to respond appropriately to users’ emotional states, thereby strengthening the therapeutic alliance and improving intervention outcomes [16,19].

Conversational agents that incorporate both voice and text modalities demonstrated superior effectiveness compared to those relying solely on textual interaction, as supported by significant improvements in mental health metrics in several included studies. These tools appear to simulate human-like interaction more authentically, which may foster greater user trust and emotional connection. For example, one study found that multimodal delivery increased engagement and emotional rapport, particularly among older adults [14]. Nonetheless, more clarity is needed regarding the magnitude and statistical significance of these improvements, which future studies should address through detailed effect size reporting.

The efficacy of AI interventions varied across demographic and clinical groups. Positive results were especially observed in clinical and subclinical populations and among older adults, although the precise mental health conditions that benefit most such as depression, anxiety, or stress require further delineation. Older adults might respond better to AI tools due to higher adherence or unique communication preferences. Importantly, individuals with more severe symptoms often preferred some degree of human involvement, indicating that AI-based tools should complement, rather than replace, traditional therapeutic approaches [23,24].

A central limitation noted across studies was the presence of considerable variability in intervention formats, outcome measures, and participant populations. This heterogeneity complicates synthesis of findings and underscores the necessity for subgroup analyses. It remains unclear whether intervention effectiveness is influenced by specific variables such as symptom type, participant age, or delivery medium. Additionally, variability in outcome measurement tools, including GAD-7, PHQ-9, and customized scales, poses challenges in interpreting cross-study consistency and comparability [2].

Further methodological concerns include small sample sizes and lack of long-term follow-up in many studies, which reduce confidence in the reliability and sustainability of intervention outcomes. The absence of longitudinal data precludes conclusions regarding relapse prevention and ongoing therapeutic impact. Many studies disproportionately focused on university students or clinical cohorts in high-income countries, limiting the generalizability of findings to more diverse, global populations. Representation from varied cultural, economic, and geographical backgrounds was limited, raising concerns regarding the universal applicability of these interventions [6]. This review also faced several procedural limitations. Language bias may have been introduced by including only English-language studies. The exclusion of gray literature and unpublished research raises the possibility of publication bias, potentially overstating intervention efficacy. Manual screening and data extraction, although performed independently by multiple reviewers, could introduce subjective interpretation and procedural inefficiencies.

Future research should prioritize large-scale randomized controlled trials involving diverse and representative populations to ensure generalizability. Longitudinal designs are essential for evaluating the durability of psychological benefits. Studies should also investigate the optimal combination of human and AI interaction. Rather than replacing clinicians, AI interventions can act as scalable, accessible adjuncts that enhance traditional therapy delivery. Additional investigation into the specific therapeutic mechanisms such as empathy, personalization, and timing of responses that drive clinical improvement is warranted [3].

Ethical and regulatory issues demand deeper examination. Core concerns such as privacy, data security, informed consent, and transparency are especially salient in mental health contexts. Policymakers and regulatory bodies must develop comprehensive frameworks to ensure ethical deployment of AI-based mental health tools. Equitable access should be a priority, particularly for underserved and low-resource populations, necessitating cross-sector collaboration among developers, healthcare providers, and public health institutions [10].

## Conclusions

AI-based conversational agents (CAs) hold considerable promise in alleviating psychological distress and promoting mental well-being, particularly when implemented with multimodal communication and personalized interaction. These tools have demonstrated effectiveness across clinical and subclinical populations, with especially positive outcomes observed among older adults. Multimodal systems that integrate text and voice have been shown to improve user engagement and therapeutic alliance, making them more effective than text-only formats. Despite these encouraging findings, inconsistencies in their impact on broader psychological well-being and the lack of long-term follow-up data underscore the need for further investigation. Future research should prioritize rigorous study designs that explore sustained effects, optimal delivery strategies, and the appropriate integration of AI tools alongside human support. Additionally, efforts must focus on addressing methodological limitations, ensuring inclusivity across diverse populations, and developing ethical frameworks to guide the equitable implementation of these technologies in global mental health care systems.

## Supporting information

S1 Checklist PRISMA 2020 checklist. The completed PRISMA 2020 checklist indicating where each reporting item is addressed in the manuscript.
(DOCX)

S1 Table Extracted data from included primary studies. Study-level data used in the meta-analysis (e.g., sample size, population/diagnosis, intervention, measures, effect sizes, follow-up, and risk-of-bias assessments). Column definitions are provided in the first rows; effect sizes are reported as Cohen’s *d*.
(DOCX)

S2 Table Comprehensive record of all studies identified in the literature search. Screening log listing each record with design, sample size, intervention, outcome measures, inclusion/exclusion decision, and reason for exclusion (if excluded), plus source/access information. This supports the PRISMA flow.
(DOCX)
