# Supplementary material for: Artificial intelligence as a predictive tool for mental health status: Insights from a systematic review and meta-analysis
Source: PLoS One. 2025 Sep 26;20(9):e0332207. doi: 10.1371/journal.pone.0332207 (PMC12469249; doi:10.1371/journal.pone.0332207)
Supplement: S1 Table — Study-level data used in the meta-analysis (e.g., sample size, population/diagnosis, intervention, measures, effect sizes, follow-up, and risk-of-bias assessments). Column definitions are provided in the first rows; effect sizes are reported as Cohen’s d. (DOCX) [file pone.0332207.s002.docx]

**Extracted Data from Included Primary Studies for Systematic Review and Meta-Analysis of AI-Based Mental Health Interventions**

| Study | Data Extractors | Date of Extraction | Eligibility Confirmed | Effect Size (Cohen's d) | Follow-up Duration | Risk of Bias | Inclusion Criteria | Exclusion Criteria | Source |
| --- | --- | --- | --- | --- | --- | --- | --- | --- | --- |
| Prochaska et al. (2021) | Arsalan Humayun, Ashwini M Madawana | 10/4/2024 | Yes | Anxiety: 0.62, Depression: 0.74 | 4 weeks | Low | RCT, mental health outcomes, AI tool use | Non-English, non-peer-reviewed | https://www.jmir.org/2021/3/e24850/ |
| Klos et al. (2021) | Arsalan Humayun, Ashwini M Madawana | 10/4/2024 | Yes | Anxiety: 0.68, Depression: 0.76 | 6 weeks | Low | RCT, mental health outcomes, AI tool use | Non-English, non-peer-reviewed | https://formative.jmir.org/2021/8/e20678/ |
| Ogawa et al. (2022) | Arsalan Humayun, Ashwini M Madawana | 10/4/2024 | Yes | Depression: 0.55 | 8 weeks | Low | RCT, mental health outcomes, AI tool use | Non-English, non-peer-reviewed | https://www.e-jmd.org/upload/jmd-21096.pdf |
| Romanovskyi et al. (2021) | Arsalan Humayun, Ashwini M Madawana | 10/4/2024 | Yes | Anxiety: 0.81, Depression: 0.85 | 12 weeks | Moderate | RCT, mental health outcomes, AI tool use | Non-English, non-peer-reviewed | https://ceur-ws.org/Vol-2870/paper89.pdf |
| Drouin et al. (2022) | Arsalan Humayun, Ashwini M Madawana | 10/4/2024 | Yes | Negative Affect: 0.65 | 8 weeks | Low | RCT, mental health outcomes, AI tool use | Non-English, non-peer-reviewed | https://www.researchgate.net/publication/374505266_Ethical_Tensions_in_Human-AI_Companionship_A_Dialectical_Inquiry_into_Replika |
| Liu et al. (2022) | Arsalan Humayun, Ashwini M Madawana | 10/4/2024 | Yes | Anxiety: 0.73, Depression: 0.79 | 4 weeks | Moderate | RCT, mental health outcomes, AI tool use | Non-English, non-peer-reviewed | https://www.sciencedirect.com/science/article/pii/S2214782922000021 |
