# Supplementary material for: Artificial intelligence as a predictive tool for mental health status: Insights from a systematic review and meta-analysis
Source: PLoS One. 2025 Sep 26;20(9):e0332207. doi: 10.1371/journal.pone.0332207 (PMC12469249; doi:10.1371/journal.pone.0332207)
Supplement: S2 Table — Screening log listing each record with design, sample size, intervention, outcome measures, inclusion/exclusion decision, and reason for exclusion (if excluded), plus source/access information. This supports the PRISMA flow. (DOCX) [file pone.0332207.s003.docx]

**Comprehensive Record of All Studies Identified in the Literature Search: Inclusion Decisions, Exclusion Reasons, and Source Access Information**

| **No.** | **Study** | **Study Design** | **Sample Size** | **Diagnosis** | **AI Intervention** | **Psychological Measure** | **Inclusion/Exclusion** | **Reason for Exclusion** | **Primary Source URL/Access Info** |
| --- | --- | --- | --- | --- | --- | --- | --- | --- | --- |
| 1 | Prochaska et al. (2021) | RCT | 118 | Substance Use Disorder | Woebot (education-based) | GAD-7, PHQ-8 | Included | N/A | <https://www.jmir.org/2021/3/e24850/> |
| 2 | Klos et al. (2021) | RCT | 181 | Depression & Anxiety | Tess (education-based) | GAD-7, PHQ-9 | Included | N/A | <https://formative.jmir.org/2021/8/e20678/> |
| 3 | Ogawa et al. (2022) | RCT | 20 | Parkinson’s Disease | Tele-consultation | BDI-II | Included | N/A | <https://www.e-jmd.org/upload/jmd-21096.pdf> |
| 4 | Romanovskyi et al. (2021) | RCT | 82 | Depression, Anxiety | Elomia (education-based) | PHQ-9, GAD-7, PANAS | Included | N/A | <https://ceur-ws.org/Vol-2870/paper89.pdf> |
| 5 | Drouin et al. (2022) | RCT | 417 | Psychological Well-being | Replika (social buddy) | PANAS | Included | N/A | <https://www.researchgate.net/publication/374505266_Ethical_Tensions_in_Human-AI_Companionship_A_Dialectical_Inquiry_into_Replika> |
| 6 | Liu et al. (2022) | RCT | 83 | Depression | XiaoNan (education-based) | PHQ-9, GAD-7, PANAS | Included | N/A | <https://www.sciencedirect.com/science/article/pii/S2214782922000021> |
| 7 | Lee et al. (2023) | Systematic Review | N/A | Various | Conversational Agents | Various | Excluded | Systematic Review, secondary source | N/A |
| 8 | Robinson et al. (2019) | Review | N/A | Various | Social Robots | Various | Excluded | Systematic Review, secondary source | N/A |
| 9 | Rathbone et al. (2017) | Systematic Review | N/A | Various | Mobile Apps & SMS | Various | Excluded | Systematic Review, secondary source | N/A |
| 10 | Scoglio et al. (2019) | Systematic Review | N/A | Various | Social Robots | Various | Excluded | Systematic Review, secondary source | N/A |
| 11 | Lee et al. (2021) | Review | N/A | Various | AI in Mental Health | Various | Excluded | Review article | N/A |
| 12 | Beyeler et al. (2023) | Study | N/A | Bariatric Care | Health Bot | Various | Excluded | Unrelated to mental health | N/A |
| 13 | Cuijpers et al. (2020) | Systematic Review | N/A | Depression | Psychotherapy | Various | Excluded | Systematic Review, secondary source | N/A |
| 14 | May et al. (2022) | Review | N/A | Various | Healthcare Chatbots | Various | Excluded | Review article | N/A |
| 15 | Firth et al. (2017) | Meta-analysis | N/A | Depression | Smartphone Apps | Various | Excluded | Secondary source | N/A |
| 16 | Ogunseye et al. (2022) | Study | N/A | Mental Health | AdaBoost Algorithm | Various | Excluded | Not focused on AI intervention directly | N/A |
| 17 | Olawade et al. (2024) | Review | N/A | Mental Health | AI-driven Tools | Various | Excluded | Review article | N/A |
| 18 | Vaidyam et al. (2021) | Systematic Review | N/A | Serious Mental Illness | Psychiatric Chatbots | Various | Excluded | Systematic Review, secondary source | N/A |
| 19 | He et al. (2023) | Systematic Review & Meta-analysis | N/A | Mental Health | Conversational Agents | Various | Excluded | Systematic Review, secondary source | N/A |
| 20 | Jabir et al. (2023) | Scoping Review | N/A | Mental Health | AI for Mental Health | Various | Excluded | Scoping Review | N/A |
| 21 | Nemesure et al. (2021) | Study | N/A | Mental Health | Predictive Modeling | Various | Excluded | Not AI-focused | N/A |
| 22 | Drouin et al. (2021) | Study | N/A | Various | Chatbot Interaction | Various | Excluded | Irrelevant for mental health focus | N/A |
| 23 | Tutun et al. (2023) | Study | N/A | Mental Health | AI Predictive Models | Various | Excluded | Not directly related to mental health outcomes | N/A |
| 24 | Dingler et al. (2021) | Yearbook | N/A | Health | Conversational Agents | Various | Excluded | Secondary source | N/A |
| 25 | May et al. (2022) | Systematic Review | N/A | Mental Health | Chatbots Privacy | Various | Excluded | Secondary source | N/A |
| 26 | Cuijpers et al. (2020) | Meta-analysis | N/A | Depression | Psychotherapy | Various | Excluded | Not AI-based interventions | N/A |
| 27 | Firth et al. (2017) | Meta-analysis | N/A | Depression | Smartphone Apps | Various | Excluded | General mental health intervention, not specific to AI | N/A |
| 28 | Tutun et al. (2023) | Study | N/A | Mental Health | AI Predictive Models | Various | Excluded | Not directly related to AI interventions | N/A |
| 29 | Jabir et al. (2023) | Scoping Review | N/A | Mental Health | AI for Mental Health | Various | Excluded | Not directly related to AI interventions | N/A |
| 30 | Lee et al. (2021) | Systematic Review | N/A | Mental Health | AI in Mental Health | Various | Excluded | Systematic Review, secondary source | N/A |
| 31 | Robinson et al. (2019) | Review | N/A | Various | Social Robots | Various | Excluded | Systematic Review, secondary source | N/A |
| 32 | Rathbone et al. (2017) | Systematic Review | N/A | Various | Mobile Apps & SMS | Various | Excluded | Secondary source | N/A |
| 33 | Scoglio et al. (2019) | Systematic Review | N/A | Various | Social Robots | Various | Excluded | Systematic Review, secondary source | N/A |
| 34 | Lee et al. (2021) | Review | N/A | Various | AI in Mental Health | Various | Excluded | Review article | N/A |
| 35 | Beyeler et al. (2023) | Study | N/A | Bariatric Care | Health Bot | Various | Excluded | Unrelated to mental health | N/A |
| 36 | Cuijpers et al. (2020) | Systematic Review | N/A | Depression | Psychotherapy | Various | Excluded | Systematic Review, secondary source | N/A |
| 37 | May et al. (2022) | Review | N/A | Various | Healthcare Chatbots | Various | Excluded | Review article | N/A |
| 38 | Firth et al. (2017) | Meta-analysis | N/A | Depression | Smartphone Apps | Various | Excluded | Secondary source | N/A |
| 39 | Ogunseye et al. (2022) | Study | N/A | Mental Health | AdaBoost Algorithm | Various | Excluded | Not focused on AI intervention directly | N/A |
| 40 | Olawade et al. (2024) | Review | N/A | Mental Health | AI-driven Tools | Various | Excluded | Review article | N/A |
| 41 | Vaidyam et al. (2021) | Systematic Review | N/A | Serious Mental Illness | Psychiatric Chatbots | Various | Excluded | Systematic Review, secondary source | N/A |
| 42 | He et al. (2023) | Systematic Review & Meta-analysis | N/A | Mental Health | Conversational Agents | Various | Excluded | Systematic Review, secondary source | N/A |
| 43 | Jabir et al. (2023) | Scoping Review | N/A | Mental Health | AI for Mental Health | Various | Excluded | Scoping Review | N/A |
| 44 | Nemesure et al. (2021) | Study | N/A | Mental Health | Predictive Modeling | Various | Excluded | Not AI-focused | N/A |
| 45 | Drouin et al. (2021) | Study | N/A | Various | Chatbot Interaction | Various | Excluded | Irrelevant for mental health focus | N/A |
| 46 | Tutun et al. (2023) | Study | N/A | Mental Health | AI Predictive Models | Various | Excluded | Not directly related to mental health outcomes | N/A |
| 47 | Dingler et al. (2021) | Yearbook | N/A | Health | Conversational Agents | Various | Excluded | Secondary source | N/A |
| 48 | May et al. (2022) | Systematic Review | N/A | Mental Health | Chatbots Privacy | Various | Excluded | Secondary source | N/A |
| 49 | Cuijpers et al. (2020) | Meta-analysis | N/A | Depression | Psychotherapy | Various | Excluded | Not AI-based interventions | N/A |
| 50 | Firth et al. (2017) | Meta-analysis | N/A | Depression | Smartphone Apps | Various | Excluded | General mental health intervention, not specific to AI | N/A |
| 51 | Tutun et al. (2023) | Study | N/A | Mental Health | AI Predictive Models | Various | Excluded | Not directly related to AI interventions | N/A |
| 52 | Jabir et al. (2023) | Scoping Review | N/A | Mental Health | AI for Mental Health | Various | Excluded | Not directly related to AI interventions | N/A |
| 53 | Lee et al. (2021) | Systematic Review | N/A | Mental Health | AI in Mental Health | Various | Excluded | Systematic Review, secondary source | N/A |
| 54 | Prochaska et al. (2021) | RCT | 118 | Substance Use Disorder | Woebot (education-based) | GAD-7, PHQ-8 | Included | N/A | Link |
| 55 | Klos et al. (2021) | RCT | 181 | Depression & Anxiety | Tess (education-based) | GAD-7, PHQ-9 | Included | N/A | Link |
| 56 | Ogawa et al. (2022) | RCT | 20 | Parkinson’s Disease | Tele-consultation | BDI-II | Included | N/A | Link |
| 57 | Romanovskyi et al. (2021) | RCT | 82 | Depression, Anxiety | Elomia (education-based) | PHQ-9, GAD-7, PANAS | Included | N/A | Link |
| 58 | Drouin et al. (2022) | RCT | 417 | Psychological Well-being | Replika (social buddy) | PANAS | Included | N/A | Link |
| 59 | Liu et al. (2022) | RCT | 83 | Depression | XiaoNan (education-based) | PHQ-9, GAD-7, PANAS | Included | N/A | Link |
| 60 | Robinson et al. (2019) | Review | N/A | Various | Social Robots | Various | Excluded | Systematic Review, secondary source | N/A |
| 61 | Rathbone et al. (2017) | Systematic Review | N/A | Various | Mobile Apps & SMS | Various | Excluded | Secondary source | N/A |
| 62 | Scoglio et al. (2019) | Systematic Review | N/A | Various | Social Robots | Various | Excluded | Systematic Review, secondary source | N/A |
| 63 | Lee et al. (2021) | Review | N/A | Various | AI in Mental Health | Various | Excluded | Review article | N/A |
| 64 | Beyeler et al. (2023) | Study | N/A | Bariatric Care | Health Bot | Various | Excluded | Unrelated to mental health | N/A |
| 65 | Cuijpers et al. (2020) | Systematic Review | N/A | Depression | Psychotherapy | Various | Excluded | Systematic Review, secondary source | N/A |
| 66 | May et al. (2022) | Review | N/A | Various | Healthcare Chatbots | Various | Excluded | Review article | N/A |
| 67 | Firth et al. (2017) | Meta-analysis | N/A | Depression | Smartphone Apps | Various | Excluded | Secondary source | N/A |
| 68 | Ogunseye et al. (2022) | Study | N/A | Mental Health | AdaBoost Algorithm | Various | Excluded | Not focused on AI intervention directly | N/A |
| 69 | Olawade et al. (2024) | Review | N/A | Mental Health | AI-driven Tools | Various | Excluded | Review article | N/A |
| 70 | Vaidyam et al. (2021) | Systematic Review | N/A | Serious Mental Illness | Psychiatric Chatbots | Various | Excluded | Systematic Review, secondary source | N/A |
| 71 | He et al. (2023) | Systematic Review & Meta-analysis | N/A | Mental Health | Conversational Agents | Various | Excluded | Systematic Review, secondary source | N/A |
| 72 | Jabir et al. (2023) | Scoping Review | N/A | Mental Health | AI for Mental Health | Various | Excluded | Scoping Review | N/A |
| 73 | Nemesure et al. (2021) | Study | N/A | Mental Health | Predictive Modeling | Various | Excluded | Not AI-focused | N/A |
| 74 | Drouin et al. (2021) | Study | N/A | Various | Chatbot Interaction | Various | Excluded | Irrelevant for mental health focus | N/A |
| 75 | Tutun et al. (2023) | Study | N/A | Mental Health | AI Predictive Models | Various | Excluded | Not directly related to mental health outcomes | N/A |
| 76 | Dingler et al. (2021) | Yearbook | N/A | Health | Conversational Agents | Various | Excluded | Secondary source | N/A |
| 77 | May et al. (2022) | Systematic Review | N/A | Mental Health | Chatbots Privacy | Various | Excluded | Secondary source | N/A |
| 78 | Cuijpers et al. (2020) | Meta-analysis | N/A | Depression | Psychotherapy | Various | Excluded | Not AI-based interventions | N/A |
